# P-839. Clinical Diagnostic Performance of Droplet Digital PCR for Pathogen Detection in Patients with *Escherichia coli* Bloodstream Infections

**DOI:** 10.1093/ofid/ofae631.1031

**Published:** 2025-01-29

**Authors:** Hiroki Kitagawa, Masato Kojima, Kayoko Tadera, Keitaro Omori, Toshihito Nomura, Norifumi Shigemoto, Hiroki Ohge

**Affiliations:** Hiroshima University Hospital, Hiroshima, Hiroshima, Japan; Hiroshima University, Hiroshima, Hiroshima, Japan; Hiroshima University Hospital, Hiroshima, Hiroshima, Japan; Hiroshima University Hospital, Hiroshima, Hiroshima, Japan; Hiroshima University Hospital, Hiroshima, Hiroshima, Japan; Hiroshima University Hospital, Hiroshima, Hiroshima, Japan; Hiroshima University Hospital, Hiroshima, Hiroshima, Japan

## Abstract

**Background:**

Rapid diagnosis and early administration of appropriate antimicrobials are crucial to improve the prognosis and decrease the mortality of patients with bacterial bloodstream infections (BSIs). Droplet digital PCR (ddPCR) is a new tool for pathogen detection in BSIs. This study aimed to examine the sensitivity of ddPCR and explore the association between bacterial DNA load in whole blood and time-to-positivity (TTP) of blood cultures (BCs) in patients with *Escherichia coli* BSIs.

Relationship between E. coli DNA load in whole blood and time-to-positivity of blood cultures

**Figure:**
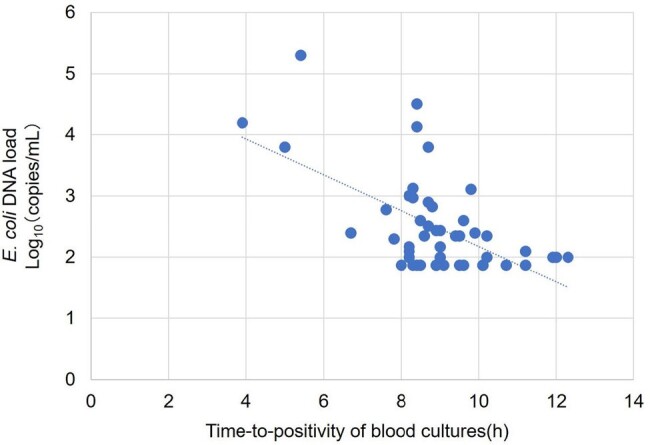

**Methods:**

This prospective study included 59 patients with *E. coli* BSIs confirmed using BCs enrolled at the Hiroshima University Hospital between June, 2023 and April, 2024. The *E. coli* DNA load in whole blood, which was simultaneously obtained from two sets of BCs, was evaluated using ddPCR. For each patient, only the TTP of the first positive BC obtained using the BacT/ALERT Virtuo System was used for analysis.

**Results:**

The median TTP was 9.0 (IQR: 8.3–10.2) h, and 15.2% (9/59) of the patients exhibited septic shock during BC sampling. In 83.1% (49/59) of the whole blood samples, *E. coli* DNA was detected via ddPCR. Patients with positive ddPCR results exhibited significantly shorter TTP than those with negative ddPCR results (median TTP: 8.8 vs. 10.5 h; p < 0.001). The rate of positive results for both sets of BCs was significantly higher in patients with positive ddPCR results than in those with negative ddPCR results (87.8 vs. 40.0%, p = 0.003). The *E. coli* DNA load was 75–200,475 copies/mL. Moreover, ddPCR revealed a significant correlation between the *E. coli* DNA load in whole blood and TTP (r: –0.58; 95% confidence interval: –0.74 to –0.36; p < 0.001). All patients with septic shock exhibited positive ddPCR results and significantly higher *E. coli* DNA loads in whole blood than those without septic shock (median *E. coli* DNA load: 6,375 vs. 113 copies/mL; p < 0.001).

**Conclusion:**

Overall, ddPCR effectively detected *E. coli* DNA in the whole blood of patients with septic shock with a relatively short TTP. Notably, *E. coli* DNA load was associated with the infection severity of the patient. Further optimization of the bacterial DNA extraction protocol from blood and reaction systems and conditions can enhance the sensitivity of ddPCR.

**Disclosures:**

**All Authors**: No reported disclosures

